# Melatonin: A Review of the Evidence for Use in Hospital Settings

**DOI:** 10.1002/prp2.70059

**Published:** 2025-01-22

**Authors:** Josephine A. Adattini, Carly Wills, Jennifer H. Martin

**Affiliations:** ^1^ Medication Safety (Formulary) Clinical Excellence Commission Sydney New South Wales Australia; ^2^ School of Medicine University of Newcastle Newcastle New South Wales Australia

## Abstract

New onset insomnia is often experienced by patients during hospitalization due to environmental disruptions, pain and increased patient care activities. Patient distress arising from poor sleep quality and quantity often results in the prescribing of hypnotics. Melatonin use in hospital settings is common and is increasingly used for off label indications including primary insomnia in those aged < 55 years, prevention of delirium and to facilitate benzodiazepine discontinuation. A literature review was conducted to evaluate the efficacy, effectiveness, safety, tolerability, and cost‐effectiveness of melatonin for various off‐label indications in inpatient hospital settings. The review found limited high quality evidence demonstrating a clinically meaningful benefit from melatonin in improving sleep, delirium, or facilitating benzodiazepine discontinuation in the inpatient setting. Study findings were inconsistent, and those that did show statistical improvement were of uncertain clinical benefit. The review also found a paucity of data on the safety of melatonin when used in hospitalized patients, and no evidence to support cost‐effectiveness. Non‐pharmacological interventions are recommended as first‐line treatment of insomnia and for the prevention of delirium in inpatient settings. The use of interventions without evidence for efficacy or effectiveness is contrary to the quality use of medicines principles in Australia's National Medicines Policy. Context‐specific evidence on the efficacy and effectiveness of a medicine should guide clinician decision‐making and prescribing, to improve the quality use of medicines.

## 1 Background

Insomnia disorder is defined as the dissatisfaction with sleep quantity or quality with complaints of difficulty initiating sleep (sleep onset latency) and/or maintaining sleep (sleep maintenance) [1]. Sleep problems are often accompanied by significant distress and impairment of daytime functioning, both social and occupational, which have been associated with reduced quality of life [1, 2]. People with insomnia have been shown to have higher rates of anxiety and depression [3], increased infectious/inflammatory pathologies [4], increased pain [5], insulin resistance [6], increased incidence of falls [7] and increased overall mortality [8].

New onset insomnia is often experienced in hospital, with patients reporting poor sleep quality and quantity due to environmental disruptions (e.g., noise and brightness) and/or increased patient care activities and monitoring as part of their care (e.g., blood draws and vital checks) [9]. Newly commenced medication adverse effects, illness, anxiety, or pain are also possible contributors to sleep problems whilst in hospital [9]. The extent of sleep disruption varies according to ward location (e.g., greater disruption reported by patients in the intensive care unit [ICU]), type of hospital accommodation (e.g., multi‐patient room), type of intervention (e.g., reduced restorative sleep is reported post‐surgery), type of illness (e.g., higher sleep disturbance in people with poorly controlled pain, chronic obstructive pulmonary disease, and heart failure), and prior diagnosis of a sleep disorder [10].

Often, hypnotics (i.e., benzodiazepines, Z‐drugs, and melatonin) are trialed in hospital to help induce sleep. Whilst they can reduce latency, they do not improve the quality of sleep, nor treat the underlying cause. They also have risks of significant harm, particularly if continued post discharge.

Benzodiazepines have been associated with an increased risk of falls, fractures, delirium, and cognitive impairment [2, 9, 11, 12, 13]. Benzodiazepines are also associated with dependence and misuse [14], with their risk for diversion and overdose/accident‐related mortality posing a growing public health concern in Australia [15]. Over the past two decades in Australia, benzodiazepines have remained the largest contributor to drug‐induced deaths, present in 44% (or 744) of drug‐induced deaths in 2021 [15]. Furthermore, 46% (or 3800) of drug‐related hospitalizations in 2021–2022 were related to benzodiazepines, and 90% of benzodiazepine‐related ambulance attendances in 2021 resulted in transfer to hospital [15].

Z‐drugs (e.g., zolpidem and zopiclone) have been marketed as a safer alternative to benzodiazepines for the management of insomnia. However, a recent network meta‐analysis demonstrated no significant differences between Z‐drugs and benzodiazepines (short and intermediate acting) in safety (incidence of adverse events) and tolerability (discontinuation due to adverse events) outcomes in the management of insomnia in adults [16].

Z‐drugs carry their own unique side effect profile (dangerous sleep behaviors, falls, head injuries) and also have clinical concerns of misuse, dependence and overdose [17].

Melatonin is frequently prescribed as an alternative to benzodiazepines and Z‐drugs for the treatment of insomnia, due to its side effect profile and reduced risk for dependence and harm. In Australia, melatonin 2 mg prolonged release (PR) tablets are Therapeutic Goods Administration (TGA) registered for use as monotherapy for the short‐term treatment of primary insomnia characterised by poor quality of sleep in patients who are aged 55 or over [18]. Melatonin 1 and 5 mg PR tablets are TGA registered for the treatment of insomnia in children and adolescents aged 2–18 with autism spectrum disorder and/or Smith‐Magenis syndrome, where sleep hygiene measures have been insufficient [19]. The use of these melatonin formulations in other indications is considered off‐label.

A recent review of inpatient prescribing practices in NSW Health has identified several off‐label uses of melatonin across NSW hospitals, including:

- Management of insomnia in inpatients receiving a palliative approach to care.
- Prevention of delirium in geriatric inpatients.
- Prevention of delirium in patients in intensive care settings.
- Benzodiazepine tapering/discontinuation in inpatients with benzodiazepine dependence.
- Management of insomnia in adult inpatients who have failed non‐pharmacological strategies.
- Management of primary insomnia in pediatric/adolescent inpatients.
- Management of insomnia in inpatients with psychiatric disorders.
- Management of insomnia in inpatients with substance use disorders.

Whilst there is some evidence that short‐term melatonin modestly improves sleep quality in healthy adults aged at least 55 years with primary insomnia, this benefit is uncertain in hospital settings where sleep distress is contributed to by the hospital environment and patient illness. It is notable that in the pivotal trials supporting the TGA registration of melatonin 2 mg PR for use in primary insomnia, a substantial proportion (74%) experienced no improvement in sleep quality [20]. Additionally, people with severe neurological, neurosurgical, and psychiatric conditions, those taking central nervous system stimulants, and people aged under 55 years were not represented in the clinical trial cohort [20]. Outcomes from controlled clinical trials may not apply to real‐world patients in hospital settings, where pre‐existing comorbidities and multiple concomitant medicines are common.

Noting the widespread use of melatonin, a literature review was undertaken to assess the available evidence on the efficacy, effectiveness, safety, tolerability, and cost‐effectiveness of melatonin for management of new‐onset insomnia, prevention of delirium and facilitation of benzodiazepine discontinuation in inpatient settings, to support clinicians and governance bodies in the decision to include or not include melatonin on hospital formularies. We have used a combination of controlled clinical trial data and data from real‐world clinical settings in our narrative review on the use of melatonin in an inpatient setting, to offer a broader perspective on melatonin's effectiveness and tolerability in patients with various comorbidities and demographic characteristics in uncontrolled settings.

## 2 Methods

The Council of Australian Therapeutic Advisory Groups (CATAG) guiding principles for the quality use of off‐label medicines provide a structured framework to evaluate medicines that are prescribed off‐label [21]. As per CATAG guiding principles, the routine use of an off‐label medicine is only justified when there is “high” quality evidence supporting the safe, efficacious, and cost‐effective use of the medicine and an overall favorable benefit: harm ratio for the intended clinical use and population [21].

A critical evaluation of evidence in published literature was conducted in August 2023. We searched the MEDLINE and PubMed databases to identify relevant articles published from 1999 to July 2023 evaluating the efficacy, safety, and cost‐effectiveness of melatonin in various inpatient settings. The off‐label indications for investigation were identified from requests by healthcare practitioners in NSW. The keywords used during the database searches were specific to each investigated indication, and included combinations of the keywords “melatonin”, “inpatients”, “hospital”, "intensive care unit", “insomnia”, “sleep quality”, “sleep disturbance”, “cost‐effectiveness”, “efficacy”, “effectiveness”, “safety”, “tolerability”, “palliative care”, “delirium”, “benzodiazepine discontinuation”, “paediatric”, “adolescent”, “children”, “psychiatric disorders”, "substance use disorders", and “substance addiction”. We also identified relevant guidelines and reviews, including the guidelines from the NSW Therapeutic Advisory Group [10], Australian Commission on Safety and Quality in Health Care [22], Australasian Sleep Association [23], The Royal Australian College of General Practitioners [24], European Sleep Research Society [25] and American Academy of Sleep Medicine [26].

The quality (certainty) of evidence from relevant studies was evaluated by considering the risk of bias, imprecision, inconsistency, indirectness, and publication bias. Levels of evidence (study design used, as an indicator of the degree of bias which has been eliminated by design) were categorized as per NHMRC guidance How to use the evidence: assessment and application of scientific evidence [27]. The size of the effect (in particular the clinical significance) and relevance of the outcome measure were other important factors considered in the evaluation of evidence.

## 3 Review of Evidence on the Efficacy, Effectiveness, Safety, Tolerability and Cost‐Effectiveness of Melatonin in Inpatient Settings

There is a lack of high‐quality evidence supporting the efficacious, safe, and cost‐effective use of melatonin in inpatient settings. A comprehensive review of published literature (1999–July 2023) found:

- Low quality (low certainty) evidence showing a lack of clinically meaningful benefit with melatonin in improving sleep in adult inpatients receiving a palliative approach to care, in preventing delirium in geriatric and intensive care inpatient settings, and in facilitating benzodiazepine discontinuation in inpatients with benzodiazepine dependence. Evidence for a clinically effective benefit in improving sleep in adult and pediatric/adolescent inpatients with primary insomnia, and in inpatients with psychiatric disorders or substance use disorders, was inconsistent and uncertain.

As the data include both clinical trials and observational studies, we have summarized the evidence for both efficacy and effectiveness for each clinical area in Table 1, with detailed evaluation of relevant literature for each clinical area presented in Data S1.

**TABLE 1:** Summary of evidence for efficacy and effectiveness of melatonin by individual clinical inpatient areas.

Clinical area	Summary of available evidence	Considerations and additional information in evaluation of evidence
Management of insomnia in inpatients receiving a palliative approach to care	Melatonin use did not improve sleep quality in adult inpatients receiving a palliative approach to care compared to placebo or standard of care (zolpidem, bright white light therapy) [28, 29, 30]	Melatonin did not improve other symptoms including fatigue, pain, loss of appetite, or quality of life compared to placebo [28, 29, 30] No studies compare outcomes to benzodiazepines or non‐pharmacological interventions such as cognitive behavioral therapy Level I–II evidence, low‐moderate certainty/quality (high heterogeneity, some concerns of bias)
Prevention of delirium in geriatric inpatients	Melatonin use did not reduce the incidence of delirium in geriatric inpatients compared to placebo [31, 32, 33]	No beneficial effects on secondary outcomes including delirium duration, length of hospital stay, or mortality [31, 33] In studies showing an efficacy benefit, both melatonin and ramelteon were evaluated together [31, 34, 35]. Subgroup analysis was not conducted by drug type in some studies [34, 35] Where subgroup analysis by drug was completed, melatonin did not show benefit in reducing delirium compared with ramelteon [31] Pre‐disposing risk factors for delirium (e.g., pre‐existing cognitive impairment, medications, and clinical characteristics of patients) were not accounted for, introducing confounding bias No comparisons to standard of care Level I‐II evidence, low‐moderate certainty/quality (high heterogeneity, some concerns of bias)
Prevention of delirium in patients in intensive care settings	Melatonin use in patients in intensive care settings did not reduce the incidence of delirium compared to placebo [33, 36, 37, 38, 39]	A multicenter randomized controlled trial across 12 Australian ICUs found that enteral administration of melatonin (4 mg every night for 14 days) did not reduce the prevalence of delirium, ICU length of stay, hospital length of stay, mortality, nor the quality or quantity of sleep compared to placebo [37] In a study showing an efficacy benefit, both melatonin and ramelteon were evaluated together [31] Subgroup analysis was not conducted by drug type [31] Pre‐disposing risk factors for delirium (e.g., pre‐existing cognitive impairment, medications, and clinical characteristics of patients) were not accounted for, introducing confounding bias No comparisons to standard of care Level I–III evidence, low‐moderate certainty/quality (high heterogeneity, some concerns of bias)
To facilitate benzodiazepine tapering/discontinuation in inpatients with benzodiazepine dependence	Melatonin use in adults with benzodiazepine dependence did not improve benzodiazepine discontinuation rates or withdrawal symptoms compared to placebo [40, 41, 42, 43, 44]	Inconsistent evidence on the effect of melatonin on sleep quality during benzodiazepine tapering/discontinuation [40, 41, 42, 45] Level I–III evidence, low‐moderate certainty/quality (high heterogeneity, some concerns of bias)
Management of insomnia in adult inpatients	There is limited and inconsistent evidence on the benefit of melatonin in improving sleep in adult inpatients with new‐onset insomnia compared to placebo [25, 26, 46]	Study findings are inconsistent. Several studies found no improvement in sleep parameters with melatonin use in the inpatient setting [37, 38, 47, 48, 49] Some studies showing a benefit grouped melatonin and ramelteon together for the efficacy outcome and it is unclear which drug the benefit was attributed to [31, 50] In studies that did show statistical improvements in sleep, the benefit was small and of questionable clinical significance [50, 51] No comparisons to standard of care (including other pharmacotherapies or non‐pharmacological interventions) Level I–III evidence, low certainty/quality (high heterogeneity, bias, indirectness, imprecision)
Management of primary insomnia in pediatric/adolescent inpatients	There is limited and inconsistent evidence on the benefit of melatonin in improving sleep in pediatrics/adolescent inpatients with primary insomnia	Paucity of data in inpatient hospital settings with new onset insomnia. Most studies were in adolescents with primary chronic insomnia in outpatient settings Study findings are inconsistent. In studies that do show improvement, the benefit was small and of questionable clinical significance [52, 53] No comparisons to standard of care (including other pharmacotherapies or non‐pharmacological interventions) Level I–II evidence, low certainty/quality (high heterogeneity, bias, indirectness, and imprecision)
Management of insomnia in inpatients with psychiatric disorders	There is limited and inconsistent evidence on the benefit of melatonin in improving sleep in adult inpatients with psychiatric disorders	Paucity of data in inpatient hospital settings. Most studies were in outpatient settings Study findings are inconsistent. Some studies found no improvement in sleep parameters with melatonin in patients with psychiatric disorders [54, 55, 56]. In studies that do show improvement, study design was suboptimal (e.g., small sample size, lack of a control group, and use of subjective outcome measures) [56, 57, 58, 59, 60] Level II–IV evidence, low certainty/quality (high heterogeneity, bias, indirectness, imprecision)
Management of insomnia in inpatients with substance use disorders	There is insufficient evidence to establish the role of melatonin in improving sleep quality among inpatients with substance use disorders. Available evidence of benefit is inconsistent	Study findings in benzodiazepine dependence and opioid addiction are inconsistent. Some studies observed no benefit with melatonin on sleep outcomes [40, 45] or withdrawal symptoms [41, 43, 44, 61] in patients withdrawing from opioids or benzodiazepines. Others reported improved sleep quality with melatonin in opioid or benzodiazepine dependence [45, 62, 63, 64] Paucity of data in nicotine addiction or alcohol use disorder Level II–IV evidence, low certainty/quality (high heterogeneity, bias, indirectness, and imprecision)

II Limited data on the safety and tolerability of melatonin when used in hospitalized patients.

Although short‐term use of melatonin has been shown in pivotal clinical trials to be well tolerated and very rarely associated with serious adverse events when used within its registered indications [65, 66], there is limited data on the safety and tolerability of melatonin in real‐world hospitalized patients with acute illness. Most studies did not report on adverse events with melatonin use, and those that did differed in the classification and reporting of adverse events. Adverse event outcomes reported in studies evaluating melatonin use in inpatient hospital settings is presented in Data S1.

III Paucity of published studies evaluating the cost‐effectiveness of melatonin in inpatient settings.
IV Limited information about comparative efficacy, effectiveness, safety, tolerability, and cost‐effectiveness between melatonin and available therapeutic alternatives, with no evidence showing a demonstrated advantage over alternatives in the relevant population and for the intended use.

## 4 Discussion

Melatonin use in hospital settings is common; however, there is a paucity of high‐quality evidence in published literature supporting its safe, efficacious, and cost‐effective use in new‐onset insomnia, delirium prevention, and benzodiazepine discontinuation in inpatient hospital settings. The use of pharmacological interventions without quality evidence for efficacy or effectiveness (e.g., the use of melatonin as a “safe placebo”) is contrary to the quality use of medicines principles in the Australia's National Medicines Policy. The National Medicines Policy also highlights that context‐specific evidence on efficacy, effectiveness, and safety is imperative in guiding clinical decision‐making focused on delivering and achieving positive health outcomes. Findings from this review have since informed decisions regarding approval pathways on initiating melatonin for inpatients in NSW Health facilities [67].

Evidence for a lack of clinically meaningful benefit of melatonin in inpatient settings is consistent with the international guidance for use of melatonin in outpatients setting. A 2017 Clinical Practice Guideline by the American Academy of Sleep Medicine for the Pharmacologic Treatment of Chronic Insomnia in Adults recommended that clinicians not use melatonin as a treatment for sleep onset or sleep maintenance insomnia (versus no treatment) in adults [26]. The 2017 European Insomnia Guideline recommends that melatonin is not generally recommended for the treatment of insomnia because of low efficacy [25].

This review is unique in that it evaluates evidence and provides guidance for melatonin use in inpatient settings, where patient environment, medications and comorbidity status may differ substantial from their state post‐discharge in the outpatient setting.

### 4.1 Limited Quality Data on the Effectiveness/Efficacy of Melatonin in Inpatient Hospital Settings

Most studies evaluating the efficacy/effectiveness of melatonin in inpatient settings were of low quality (low certainty), due to inconsistency, indirectness, imprecision, and bias.

There was significant heterogeneity (inconsistency) between studies in population characteristics, intervention (melatonin dose, form, and duration), and outcome measures (methods used to measure sleep, benzodiazepine discontinuation protocols, methods used to measure delirium, and classification and reporting of adverse events), limiting the generalizability of results and synthesis of findings. The use of subjective sleep outcome measures (rather than objective assessments of sleep) introduced indirectness. In many cases, improvement in efficacy outcomes was small and of uncertain clinical relevance. Small sample size and uncertain specified margin of clinical significance introduced imprecision. Observational single‐arm studies lacking a comparator to either standard of care or placebo reduced confidence in establishing causation and introduced confounding bias. Furthermore, most studies evaluating the efficacy/effectiveness of melatonin for delirium prevention in inpatient settings did not account for confounding variables (e.g., pre‐existing cognitive impairment, medications, and clinical characteristics of patients), increasing the risk of bias.

#### 4.1.1 Potential Confounding Bias: Pharmacological Variability in Melatonin Agonists

Confounding bias was also introduced when studies grouped melatonin and ramelteon together, with no subgroup analysis of outcomes by intervention/drug. Whilst both are melatonin receptor agonists, they exhibit distinct pharmacokinetic and pharmacodynamic properties. For example, in vitro studies have shown that ramelteon (not a TGA‐registered medicine) has three‐ to five‐fold greater binding affinity for MT1 and MT2 receptors than exogenous prolonged‐release melatonin, and 16.9 times higher MT2 receptor‐stimulating potency than melatonin [68]. These pharmacological properties suggest that these two agents should be studied separately. Grouping both melatonin and ramelteon may have potentially suggested an efficacy benefit in some studies that is more likely attributed to ramelteon alone.

### 4.2 Limited Quality Data on the Safety/Tolerability of Melatonin in Inpatient Hospital Settings

Whilst short‐term use of melatonin is considered well‐tolerated in adults aged > 55 with primary insomnia, the tolerability of melatonin in real‐world hospitalized patients is uncertain because of a lack of reporting of safety outcomes in most studies, and limitations in methodological features in studies which did report safety outcomes. Real‐world patients in hospital do not necessarily reflect those studied in clinical trials, due to differences in physiology (secondary to acute illness), concomitant medicines and comorbidity burden.

In studies that do explore adverse events with melatonin use in hospital, significant heterogeneity (inconsistency) in the definition and reporting of adverse events makes it difficult to draw definitive conclusions. Heterogeneity in melatonin dose/duration and in population characteristics (demographic and disease characteristics, concomitant medicine usage, clinical setting) also hindered generalizability of safety findings. Data collected were the short‐term use of melatonin, with none of the included studies exploring the long‐term implications of melatonin initiation in various patient groups. There is some low‐quality evidence that exogenous melatonin may alter pubertal timing in animals [69]. Longitudinal studies in humans suggested that long‐term melatonin use (follow‐up of 3.8 years) might be linked to a delay in puberty onset [70]; however, the determination of puberty delay was subjective (perceived) and not consistent with findings from other studies [69, 71]. The current scarcity of safety data calls for further assessment of adverse events in real‐world patients in inpatient hospital settings.

We acknowledge the limitations of not using the Cochrane or similar methods to undertake a systematic review and meta‐analysis. However, with widespread prescription of melatonin for a myriad of symptoms, in different populations and at different doses, we felt it prudent to share our literature review findings and provide an overview of the current evidence base in hospital settings. We find it interesting that there is widespread prescription and public funding for such an agent in the inpatient hospital setting given the absence of evidence for benefit.

### 4.3 Paucity of Evidence Evaluating Melatonin Cost‐Effectiveness in Inpatient Hospital Settings

This review also found no evidence to support cost‐effectiveness of melatonin for use in off‐label inpatient settings. An economic evaluation, by definition, is the *comparative analysis of alternative courses of action in terms of both their costs and consequences* [72]. Therefore, for cost‐effectiveness analyses to be valid, the underlying measure of effectiveness needs to be understood for both the intervention and comparator. However, a comparator arm was lacking in most studies in the reviewed off‐label inpatient settings, and the evidence for melatonin's effectiveness was either uncertain/inconsistent or showing no efficacy benefit. For routine and widespread use of melatonin in an off‐label setting, additional information about cost‐effectiveness is required (CATAG guiding principles).

### 4.4 Non‐Pharmacological Interventions Should Be First‐Line for Management of New‐Onset Insomnia and Prevention of New‐Onset Delirium in Inpatient Hospital Settings

Non‐pharmacological interventions are recommended as first‐line treatment of insomnia [10, 22, 25, 26] and should be provided for all patients in hospital [10]. Many factors known to disrupt sleep in hospitalized patients are modifiable (e.g., environmental noise and awakening by hospital staff) [73, 74]. The NSW Therapeutic Advisory Group guidance Getting it right for sleep at night outlines evidence‐based strategies for managing sleep disturbances or insomnia in hospitalized adults [10]. Sleep disturbance in acute care hospitals is most effectively prevented by changing the delivery of care and creating a ward environment conducive to rest and sleep [10]. Individual patient‐related strategies (e.g., cognitive behavioral therapy, recognize and manage contributing medicines or illness/comorbid conditions such as pain, breathlessness, nausea, and vomiting) should also be explored [10]. New onset of insomnia in hospital is usually short term, with most patients showing resolution of insomnia within 2 weeks of discharge [9].

The Australian Commission on Safety and Quality in Health Care Delirium Clinical Care Standard (Quality statement 2) [22], recommends non‐pharmacological interventions to prevent delirium. These interventions include ward nursing education programs, medication reviews (including medication reconciliation before transitions of care), mobilization activities, optimization of hydration and nutrition, optimization of pain management, environmental care strategies (e.g., reducing noise, ensuring appropriate lighting, avoiding ward moves, provide sensory aids, and help orientate) and the use of non‐pharmacological measures to help promote sleep (e.g., relaxation techniques and earplugs in the ICU) [10, 22]. Additionally, the Delirium Clinical Care Standard (Quality statement 7) recommends non‐pharmacological strategies to manage behavioral and psychological symptoms in patients with delirium [22]. The Hospital Elder Life Program is an example of a model of hospital care that has demonstrated effectiveness in reducing the incidence of delirium and falls in older, hospitalized adults [75].

We acknowledge there may be difficulties with implementing non‐pharmacological approaches in hospital settings; however, we suggest that attention to simple interventions, that are relatively straightforward, can help patients sleep whilst in hospital (for example, dimming lights, reducing noise, and providing eye masks). We suggest that the use of a medicine (melatonin) without evidence of benefit, should not be used in place of these strategies.

## 5 Conclusion

There is a lack of high‐quality evidence supporting the efficacious, safe, and cost‐effective use of melatonin for the management of sleep problems, delirium prevention and benzodiazepine discontinuation in inpatient settings. Non‐pharmacological interventions are recommended as first‐line for the treatment of new‐onset insomnia and prevention of delirium in hospital settings. Clinicians should consider context‐specific evidence for efficacy/effectiveness benefits before prescribing melatonin. Further well‐designed studies demonstrating safety/tolerability and clinically meaningful benefits in specific patient groups are required before widespread recommendations for the use of melatonin in hospital settings can occur.

## Author Contributions

**Josephine A. Adattini:** conceptualization (equal); methodology (lead); writing – original draft (lead); writing – review and editing (equal). **Carly Wills:** conceptualization (equal); writing – review and editing (equal). **Jennifer H. Martin:** conceptualization (equal); supervision (lead); writing – review and editing (equal).

## Conflicts of Interest

The authors declare no conflicts of interest.

## Supporting information


Data S1.

## Data Availability

The data that supports the findings of this study are available in the supplementary material of this article.
