# Supplementary material for: Melatonin: A Review of the Evidence for Use in Hospital Settings
Source: Pharmacol Res Perspect. 2025 Jan 22;13(1):e70059. doi: 10.1002/prp2.70059 (PMC11751625; doi:10.1002/prp2.70059)
Supplement: Supplementary file 1 — Data S1. [file PRP2-13-e70059-s001.docx]

## **Evaluation of the literature on melatonin efficacy/effectiveness and safety in clinical inpatient areas**

1. ***Management of insomnia in inpatients receiving a palliative approach to care***

Summary of evidence: Melatonin use did not improve sleep quality in adult inpatients receiving a palliative approach to care compared to placebo or standard of care (zolpidem, bright white light therapy).

In a double-blind, randomised, placebo-controlled crossover trial (72 patients), oral melatonin 20 mg/day for 1 week did not improve symptoms of physical fatigue, insomnia, loss of appetite, pain, emotional function, nor overall quality of life compared to placebo in patients with advanced cancer receiving palliative treatment.(1) No serious adverse events (defined as adverse events leading to hospitalisation or death) were recorded with melatonin 20 mg/day for 1 week.(1)

A double-blind, randomised, placebo-controlled trial in 48 patients with advanced lung or gastrointestinal cancer observed no improvement in symptoms of cachexia, appetite, depression, pain nor insomnia with oral melatonin 20 mg/day for 28 days compared to placebo.(2) The incidence of adverse events and of discontinuation due to serious adverse events were comparable between melatonin and placebo groups.(2)

A systematic review of randomised controlled trials (19 studies, 2101 patients) demonstrated that melatonin had no significant effect on quality of life, sleep quality, fatigue, pain or stomatitis severity in patients with cancer.(3) Specifically, nine studies evaluated sleep quality (1128 patients), of which 7 studies compared melatonin to placebo, one study compared melatonin to zolpidem 10 mg daily and one study compared melatonin with bright white light therapy (BWLT) to BWLT alone.(3) There was significant heterogeneity between studies, with variation in intervention (melatonin daily dose varied from 2 mg to 20 mg, duration ranged from 10 days to 4 months) and outcome measures (measurement of sleep). Some studies showed concerns for bias. The safety / adverse event profile of melatonin in patients with cancer was not explored in this review.

1. ***Prevention of delirium in geriatric inpatients***

Summary of evidence: Melatonin use did not reduce the incidence of delirium in geriatric inpatients compared to placebo.

A meta-analysis (including studies up to 2017) evaluating the efficacy of melatonin and ramelteon in the prevention of delirium in postoperative hospitalised patients (mean patient age ≥ 50 years) demonstrated a reduced odds of delirium with melatonin/ramelteon compared to placebo (Odds Ratio [OR], 0.63; 95% Confidence Interval [CI], 0.47–0.87; *P* = 0.006; 6 studies, 1155 patients).(4) However, subgroup analysis was not conducted by drug type (melatonin vs. ramelteon) or hospital setting (surgical inpatient and ICU were combined), there was high heterogeneity among included studies (type of surgical procedure and melatonin dosing regimens), and predisposing risk factors for delirium (e.g., pre-existing cognitive impairment, medications) were not accounted for in the analysis. Safety outcomes were not investigated in this meta-analysis.

In a meta-analysis of randomised controlled trials (mean patient age of 67.3 years, included studies published up to 2020), melatonin/ramelteon significantly reduced the risk of delirium in hospitalised patients compared to placebo (Risk Ratio [RR], 0⋅61; 95% CI, 0⋅42–0⋅89; *P* = 0⋅009; fourteen studies; 1712 patients).(5) However, when subgroup analysis was conducted by drug type (melatonin vs. ramelteon), melatonin no longer showed beneficial effects in reducing the risk of delirium (RR, 0⋅67; 95% CI, 0⋅41–1⋅09; *P* = 0⋅11; nine studies; 1295 patients), whereas ramelteon did (RR, 0⋅51; 95% CI, 0⋅27–0⋅93; *P* = 0⋅03; five studies; 417 patients).(5) When subgroup analysis was conducted by clinical settings, melatonin/ramelteon did not significantly reduce the risk of delirium in surgical patients (RR, 0⋅51; 95% CI, 0⋅25–1⋅03; *P* = 0⋅06; six studies; 967 patients) or in medical patients (RR, 0⋅88; 95% CI, 0⋅15–5⋅31; *P* = 0⋅89; two studies; 209 patients).(5) Melatonin/ramelteon did not reduce delirium duration, length of hospital stay, and mortality.(5) Substantial heterogeneity was observed among included studies, and predisposing risk factors for delirium (e.g., pre-existing cognitive impairment, medications) were not accounted for in the analysis. Safety outcomes were not investigated in this meta-analysis.

A Cochrane review (included studies published up to 2015) assessing the effectiveness of interventions for preventing delirium in inpatients admitted to internal medicine wards found no clear evidence that melatonin (0.5 mg/day in one study, 3 mg/day in another study) or melatonin agonists (ramelteon 8 mg/day) reduce delirium incidence compared to placebo (RR, 0.41; 95% CI, 0.09–1.89; three studies, 529 patients; low‐quality evidence) in older patients (mean patient age of 84 years).(6) None of the included studies investigated adverse events with melatonin when used for preventing delirium in inpatients.

In a meta-analysis of randomised controlled trials (studies published up to 2018), melatonin (dose ranged from 0.5 mg to 50 mg/kg) did not reduce the incidence of delirium in hospitalised patients (aged > 50 years) compared to placebo (OR, 0.74; 95% CI, 0.29–1.92; seven studies, 982 patients), and did not reduce the duration of hospital length of stay (mean difference, - 1.01 days; -2.80 to 0.79; six studies, 634 patients).(7) No significant differences were noted on all-cause mortality, ICU mortality and common adverse events (nausea, dizziness, headache, paraesthesia) between melatonin/ramelteon and placebo arms.(7) Authors noted a lack of uniformity of the diagnostic criteria for delirium, varied dosage and duration of melatonin, and differences in patients’ baseline characteristics, use of concomitant medications and different clinical settings between the included trials.

A systematic review of randomised controlled trials (studies published up to 2022) of melatonin/ramelteon in post-operative delirium in patients aged over 50 years concluded a significantly reduced incidence of post-operative delirium associated with melatonin/ramelteon use (OR, 0.41; 95% CI, 0.21–0.80; *P*=0.01; 11 studies, 1244 patients), however authors did not conduct a subgroup analysis by drug type (melatonin vs. ramelteon) and there was significant heterogeneity among studies.(8) Safety outcomes were not investigated in this systematic review.

1. ***Prevention of delirium in patients in intensive care settings***

Summary of evidence: Melatonin use in patients in intensive care settings did not reduce the incidence of delirium compared to placebo**.**

A multicentre randomised controlled trial across 12 Australian intensive care units (ICUs; 847 patients, mean patient age of 62 years) found that enteral administration of melatonin (4 mg at 9 pm for 14 consecutive days) initiated within 48 hours of ICU admission did not reduce the prevalence of delirium compared to placebo (proportion of delirium-free assessments per patient was 79% with melatonin vs. 80% with placebo, *P* = 0.55).(9) There was also no significant difference in secondary outcomes including ICU length of stay (median 5 vs. 5 days, *P* = 0.14), hospital length of stay (median 14 vs. 12 days, *P* = 0.82), mortality at any time point including at 90 days (15.5% vs. 15.6%, *P* = 0.95), nor in the quantity or quality of sleep between patients receiving melatonin 4 mg and placebo.(9) No patients experienced serious adverse events (death or significant morbidity) related to melatonin.(9)

A multicentre randomised controlled trial of 203 patients admitted to ICUs showed no beneficial effect of melatonin 10 mg (administered enterally or via NG tube for 7 consecutive nights) on delirium scores compared to placebo.(10) A total of 63 patients experienced an adverse event, however this study did not delineate between the incidence of adverse events in each treatment arm and did not evaluate the likely causal relationship to the treatment.

In a meta-analysis of randomised controlled trials (studies published up to 2018), melatonin did not reduce the incidence of delirium in hospitalised patients (intensive care and non-intensive care settings) aged > 50 years compared to placebo (OR, 0.74; 95% CI, 0.29–1.92; *P* = 0.54; seven studies; 982 patients, moderate certainty).(7) In a subgroup analysis of studies conducted in ICUs, melatonin/ramelteon did not reduce the incidence of delirium in intensive care settings compared to placebo (OR, 0.74; 95% CI, 0.19–2.82; *P*=0.66; four studies; 305 patients).(7) Melatonin/ramelteon did not significantly reduce the need for sedative agents, duration of mechanical ventilation, and length of hospital stay in hospitalised patients.(7) No significant differences were noted on all-cause mortality, ICU mortality and common adverse events (nausea, dizziness, headache, paraesthesia) between melatonin/ramelteon and placebo arms.(7) Melatonin/ramelteon, however, did show benefit in decreasing ICU length of stay by a mean of 1.84 days lower compared to placebo (95% CI, -2.46 to -1.21; five studies; 411 patients).(7) It is unclear whether the observed effect was due to melatonin or ramelteon, as the analysis by clinical setting grouped both drugs (melatonin and ramelteon) together.(7) Authors noted this finding should be interpreted with caution, as predisposing risk factors for delirium/increased length of stay (e.g., pre-existing cognitive impairment, medications, clinical characteristics of patients) were not accounted for in the analysis.(7) Additionally, substantial heterogeneity was observed among included studies (due to differences in delirium screening tools/diagnostic criteria, dosage and duration of melatonin administered).(7)

In a meta-analysis of randomised controlled trials (studies published up to 2022), melatonin did not reduce the incidence of delirium in ICU patients (RR, 0.82; 95% CI, 0.57–1.17; *P* = 0.27; five studies – two overlapped with meta-analysis by Ng et al; 1537 patients).(11) Additionally, no beneficial effects were observed with melatonin and ramelteon on ICU length of stay (mean difference – 0.26; 95% CI, -0.89–0.37; *P* = 0.42; eight studies; 1453 patients) and mortality (RR, 0.85; 95% CI, 0.63–1.15; *P* = 0.30; seven studies; 1661 patients).(11) A non-significant trend for reduced duration of mechanical ventilation was observed with melatonin (mean difference – 2.80; 95% CI, -6.06–0.47; *P* = 0.09; four studies; 989 patients).(11) Safety outcomes were not investigated in this meta-analysis.

In a meta-analysis of randomised controlled trials (mean patient age 67.3 years, included studies published up to 2020), melatonin/ramelteon reduced the incidence of delirium in ICU patients compared to placebo (RR, 0⋅66; 95% CI, 0⋅50–0⋅88; *P* = 0⋅004; five studies; 469 patients).(5) However, it is unclear whether the observed effect was due to melatonin or ramelteon, as the analysis by clinical setting grouped both drugs (melatonin and ramelteon) together. Melatonin/ramelteon did not reduce delirium duration, length of hospital stay or all-cause mortality in hospitalised patients (intensive care and non-intensive care settings), or ICU length of stay, compared to placebo.(5) Substantial heterogeneity was observed among included studies, and predisposing risk factors for delirium (e.g., pre-existing cognitive impairment, medications) were not accounted for in the analysis. Safety outcomes were not investigated in this meta-analysis.

An abstract of a single-center observational cohort study (268 patients) evaluating the efficacy of melatonin in critically ill patients reported no beneficial effect of melatonin on the incidence of delirium and perceived sleep outcomes (sleep depth, sleep latency, awakenings, ability to return to sleep, sleep quality, and perception of noise) compared to patients receiving other sleep medicines (alone or combined with melatonin) or no sleep medication.(12) This study observed the highest incidence of delirium in patients receiving melatonin alone.(12) The dose and duration of melatonin, in addition to safety outcomes, were not reported.

1. ***Benzodiazepine tapering/ discontinuation in inpatients with benzodiazepine dependence.***

Summary of evidence: Melatonin use in adult inpatients with benzodiazepine dependence did not improve benzodiazepine discontinuation rates or withdrawal symptoms compared to placebo.

In a double-blind cross-over study of 61 patients enrolled at a community methadone maintenance clinic attempting benzodiazepine withdrawal, melatonin 5 mg/day for 6 weeks did not improve benzodiazepine discontinuation rate or time to benzodiazepine cessation compared to placebo, nor did it improve subjective sleep quality / perceived sleep outcomes (sleep duration, sleep disturbances, sleep latency, daytime functioning due to sleepiness, sleep efficiency, sleep quality, sleep medication use) in those who discontinued benzodiazepines.(13) Subjective sleep quality, however, did improve in patients who discontinued benzodiazepines compared to those who didn’t, regardless of the accompanying treatment (melatonin or placebo).(13) This study showed concerns for attrition bias, with a large proportion of patients lost to follow-up. Melatonin-related adverse events were not explored in this study.

A meta-analysis of randomised placebo-controlled trials (studies published up to 2014) found no effect of melatonin (PR 2 mg, IR 3 mg or IR 5 mg, daily over 4 to 6 weeks) on the odds of discontinuing benzodiazepines (OR, 0.72; 95% CI, 0.21–2.41, *P* = 0.59, four studies, 244 patients) among adults with benzodiazepine dependence (mean patient age of 64 years).(14) There were mixed effects observed with melatonin on sleep quality (four studies, 181 patients), with two trials reporting significant improvements in sleep quality measures in the melatonin arm whilst two trials did not.(14) Due to different methods for assessing sleep quality, results for sleep quality were not pooled in the meta-analysis. The small sample size contributed to a wide confidence interval and reduced precision of the point estimate of the OR for benzodiazepine discontinuation in the meta-analysis. The review did not explore safety outcomes with melatonin use in this clinical setting.

In a 2018 Cochrane review investigating pharmacological interventions to facilitate benzodiazepine discontinuation in chronic benzodiazepine users, no difference was observed in benzodiazepine discontinuation risk between melatonin (PR 2 mg/day or IR 5 mg/day for 4 to 10 weeks) and placebo (RR, 1.20; 95% CI, 0.73–1.96; *P* = 0.48, four studies, 219 patients).(15) The meta-analysis also found no beneficial effect with melatonin over placebo for self-reported sleep quality (standardised mean difference -0.31; 95% CI, -0.92–0.31; *P* = 0.33, two studies, 116 patients), and no difference in discontinuation due to adverse events (RR, 2.10; 95% CI, 0.20–22.26; *P* = 0.54; two studies, 120 patients).(15)

A single-centre randomised controlled trial in 86 patients with schizophrenia or bipolar disorder attempting long-term benzodiazepine discontinuation found no significant effect of melatonin PR 2 mg daily (compared to placebo) on mean benzodiazepine dosage at 24 weeks (8.01 mg vs. 5.72 mg diazepam equivalent; *P* = 0.20) or benzodiazepine discontinuation rate (OR, 0.64; 95% CI, 0.26–1.56; *P* = 0.32).(16) Melatonin also showed no effect on benzodiazepine withdrawal symptoms.(16) The rate of occurrence of adverse events (including ECG and laboratory abnormalities), serious adverse events, and discontinuation due to adverse events were comparable between the melatonin and placebo groups.(16) Secondary analyses of this cohort found no beneficial effects with melatonin PR 2 mg/day on cognitive functioning, quality of life, subjective well-being and psychosocial functioning during benzodiazepine withdrawal over placebo.(17) Further analysis was conducted on a subsample of 23 patients undergoing sleep recordings and 55 patients participating in subjective sleep quality ratings.(18) Despite showing improved self-reported sleep quality, melatonin PR 2 mg/day for 24 weeks did not show beneficial effects on objective sleep efficiency outcomes (i.e., total sleep time, sleep latency, number of awakenings, REM latency) compared to placebo.(18)

A randomised placebo-controlled trial in 92 patients with primary insomnia and chronic benzodiazepine use attempting benzodiazepine discontinuation in an outpatient clinic did not show improvement in withdrawal symptoms with melatonin PR 2 mg/day during the 1 month withdrawal period.(19) A non-significant trend for increased benzodiazepine discontinuation rates were observed with melatonin compared to placebo (1-month discontinuation rate of 67% vs. 85%, *P* = 0.05; 6-month discontinuation rate 32% vs. 44%, *P* = 0.22).(19) There were no serious adverse events in either group during the withdrawal period or at follow-up.(19)

1. ***Management of insomnia in adult inpatients who have failed non-pharmacological strategies.***

Summary of evidence: There is limited and inconsistent evidence on the benefit of melatonin in improving sleep in adult inpatients with new-onset insomnia compared to placebo.

A multicentre randomised controlled trial across 12 Australian ICUs (847 patients, mean patient age of 62 years) found that enteral administration of melatonin (4 mg at 9pm for 14 consecutive days) initiated within 48 hours of ICU admission did not improve the quantity or quality of sleep compared to placebo.(9) No serious adverse events were reported in this study.

A Cochrane review (four studies published up to 2017, 151 patients) found insufficient evidence to determine whether melatonin administration was effective in improving the quality and quantity of sleep in critically ill adult patients in the ICU setting.(20) All studies compared melatonin against no agent; three were placebo‐controlled trials; and one compared melatonin with usual care.(20) The review also noted that the adverse event profile of melatonin in this setting is uncertain, with studies differing in the reporting of adverse events. Certainty of evidence was limited by sparse data (reducing precision of effect estimates), with heterogeneity in study methodology (i.e., melatonin dose, ICU sedation protocols, methods used to measure sleep) introducing inconsistency and indirectness to the outcome data.

A single centre randomised controlled trial (33 participants) in an ICU cohort found no beneficial effect of enteral melatonin (3 mg loading dose at 9 pm, followed by 0.5 mg hourly maintenance dose until 3 am via NG tube) on sleep outcomes (arousals per hour of sleep, proportion of each sleep stage, length of deep sleep) compared to placebo.(21) The review did not explore safety outcomes with melatonin use in this clinical setting.

A meta-analysis by Khaing et al. (included studies published up to 2020) reported longer total sleep (mean difference 26 mins) and less sleep disturbances (number of awakenings per night was 1 vs. 1.5) with melatonin/ramelteon compared to placebo in hospitalised patients (three studies, 242 patients).(5) However, there was high heterogeneity among studies included in the meta-analysis, and outcomes were not separated by drug type (melatonin vs. ramelteon) or hospital setting (medical inpatient and ICU were combined). Safety outcomes were not investigated in this meta-analysis.

A multicentre randomised controlled trial (203 patients) showed improved perceived sleep quality in ICU with melatonin 10 mg/day (enterally or via NG tube for 7 consecutive nights) compared to placebo, however, no significant differences were observed in perceived sleep quality on general wards nor in duration of sleep (measured by nurse assessment) in ICU and general wards compared to placebo.(10) A total of 63 patients experienced an adverse event, however this study did not delineate between the incidence of adverse events in each treatment arm and did not evaluate the likely causal relationship to the treatment.

A single centre randomised controlled trial (69 patients) found that nightly administration of melatonin 3 mg during hospitalisation did not improve sleep measurements (i.e., total sleep time per 24 hours, night-time sleep duration) in inpatients age ≥ 65 years, admitted to internal medicine wards (not ICU), compared to placebo.(22) No serious adverse events were reported in this study.(22)

A meta-analysis by Marupuru et al. (studies published up to 2021) demonstrated significantly improved objective total sleep time (mean 21 mins, 9 studies, 607 patients), objective sleep latency (mean 14 mins, 8 studies, 1110 patients) and self-reported sleep quality (10 studies), but not objective sleep efficiency (8 studies, 901 patients), with melatonin/ramelteon compared to placebo in older adults (age ≥ 50) with chronic insomnia.(23) However, results were not stratified by drug (included ramelteon) and the majority of included studies were in the outpatient setting.(23) Improvement in sleep parameters were small and of questionable clinical benefit.(23) Studies also showed concerns for bias, were of low methodological quality, and showed high heterogeneity. Safety outcomes were not investigated in this meta-analysis.

A small randomised double-blind placebo-controlled crossover study in 33 patients with sleep disturbances following traumatic brain injury observed an improvement in self-reported sleep quality and in actigraphy sleep efficiency with melatonin (2 mg MR daily for four weeks) compared to placebo, however, the improvement in objective sleep efficiency was small and no beneficial effect on sleep onset latency was observed with melatonin.(24) No serious adverse events were reported in this study,(24) however, the methodology of adverse event reporting and classification of a serious adverse event were not defined.

1. ***Management of primary insomnia in paediatric/ adolescent inpatients***

Summary of evidence: There is limited and inconsistent evidence on the benefit of melatonin in improving sleep in paediatrics/adolescent inpatients with primary insomnia.

A meta-analysis (19 studies published up to 2011) of melatonin in children and adults with primary sleep disorders reported significantly reduced sleep latency (mean difference =  7.06 minutes; 95% CI, 4.37–9.75; *P* < 0.001), increased total sleep time (mean difference  =  8.25 minutes; 95% CI, 1.74–14.75; *P* =  0.013), and improved sleep quality (mean difference = 0.22; 95% CI, 0.12–0.32; *P* < 0.001) with melatonin compared to placebo,(25) however the clinical benefit of these differences is small and likely insignificant. Studies in the meta-analysis measuring sleep latency and total sleep time showed significant heterogeneity (inconsistencies in how sleep data was obtained, form and dose of melatonin, timing of administration, duration of melatonin treatment), and had small sample sizes. Some studies had a high or unclear risk of bias. None of the studies were conducted in inpatient settings. Safety outcomes were not investigated in this meta-analysis.

A meta-analysis (8 studies published up to 2023) with 419 children and adolescents with idiopathic chronic insomnia showed a moderate increase in parent-reported total sleep time by 30 minutes (4 studies) and moderate reduction in parent-reported sleep latency by 18 minutes (3 studies).(26) However, no beneficial effects were seen with sleep efficacy (measured by actigraphy) or parent-reported daytime functioning.(26) All studies investigated the effects of melatonin in comparison to placebo, and none were conducted in inpatient settings.(26) Additionally, none of the studies provided information on serious adverse events, however the number of participants experiencing non-serious adverse events was increased with melatonin compared to placebo (relative risk 3.44; 95% CI, 1.25–9.42; 4 studies).(26)

1. ***Management of insomnia in inpatients with psychiatric disorders***

Summary of evidence: There is limited and inconsistent evidence on the benefit of melatonin in improving sleep in adult inpatients with psychiatric disorders.

Evidence for melatonin efficacy in patients with bipolar disorder is conflicting. A randomised controlled trial in 41 patients with bipolar disorder (types 1 and 2) observed no significant difference between melatonin PR 2 mg/day and placebo on self-reported sleep outcomes and actigraphy sleep outcomes (sleep onset, offset and total sleep time).(27) Small single-centre observational case series (level IV evidence, low quality) suggest beneficial effects of melatonin for symptoms of sleep disturbance in bipolar disorder, however, findings are difficult to interpret due to limitations in study designs (i.e., small sample size, lack of control group and use of subjective sleep outcome measures).(28, 29) An open-label study in 11 patients with bipolar disorder type 1 showed that adjunctive melatonin IR 3 mg/day for four weeks significantly improved self-reported total sleep time (mean sleep duration increased by 2.81 hours) and decreased self-reported manic symptom severity.(28) In a cohort of 14 patients with bipolar disorder and insomnia, melatonin IR 3 to 6 mg/day administered as an adjunctive to usual mood stabilising treatment improved subjective sleep quality and duration of sleep, with a decrease in residual depressive symptoms.(29)

Evidence for melatonin efficacy in patients with depression is conflicting. A small randomised controlled trial in 19 patients with depression observed improved self-reported sleep quality (using the Pittsburgh Sleep Quality Index) with adjunctive melatonin PR 5 - 10 mg/day for 4 weeks compared to placebo.(30) Conversely, in a more recent randomised controlled trial investigating the use of melatonin for sleep in depression, four weeks of melatonin PR 6 mg/day did not demonstrate significant improvements in self-reported sleep quality (time to sleep, number of awakenings) or objective sleep measures (sleep latency, total sleep time, sleep efficiency, number of awakenings) compared to placebo.(31)

Whilst some studies suggest potential beneficial effects of melatonin for symptoms of sleep disturbance in patients with schizophrenia in the outpatient setting, clinical significance is uncertain. In a small randomised cross-over trial in 19 patients with schizophrenia, melatonin PR 2 mg for 3 weeks improved sleep efficiency (84% *vs.* 78%, *P* = 0.038), and showed non-significant trends to improved sleep latency (by 40 minutes, *P* = 0.06) and sleep duration (by 45 minutes, *P* = 0.08) in low, but not high, efficiency sleepers compared to placebo.(32) However, the benefit observed in sleep efficiency is likely not of clinical significance. A small randomised controlled trial in 40 patients with schizophrenia demonstrated that melatonin IR 3 to 12 mg/day improved self-reported sleep quality during the initial 10 days of treatment, reduced the number of night-time awakenings (mean 0.75 vs. 1.70, *P* = 0.045) and increased sleep duration (5.7 vs. 5.4 hours, *P* = 0.021) compared to placebo, although the differences are likely not of clinical significance.(33) No benefit was observed in sleep onset latency with melatonin compared to placebo (1.14 vs. 1.11 hours, *P* = 0.38).(33)

Most literature investigating melatonin for the management of insomnia in people with attention-deficit/hyperactivity disorder (ADHD) is in children and adolescents for which there is some evidence for improvement in sleep onset latency.(34, 35) There is a paucity of data on the efficacy of melatonin for insomnia in adults with ADHD. A randomised controlled trial in 51 adults with ADHD and delayed sleep phase disorder observed that three weeks of low doses of melatonin (0.5 mg/day) advanced the circadian rhythm and reduced self-reported ADHD symptoms compared to placebo.(36) Sleep efficacy endpoint measures (e.g., sleep quality, sleep efficiency, sleep duration, sleep latency etc.) were not investigated.(36)

Most literature investigating melatonin in people with autism spectrum disorder (ASD) is in children and adolescents, for which there is strong evidence for beneficial effects in symptoms of insomnia (bedtime resistance, sleep onset latency, sleep continuity, total sleep time) and symptoms of ASD in children.(37-43) There is a paucity of data on the efficacy of melatonin for insomnia in adults with ASD. A small retrospective observational case series (level IV evidence, low quality) in 6 adults with sleep disturbances and ASD admitted to psychiatric units observed improvements in sleep duration after treatment with melatonin IR 6 mg/day for 6 months (results were not pooled).(44) The small sample size, use of an unvalidated tool for sleep outcome measurement, and lack of control/adjustment for other potentially confounding variables, are some factors which limit the interpretation of this finding.

Most studies investigating the use of melatonin in patients with psychiatric disorders did not explore the safety / tolerability of melatonin.(28, 29, 31, 32, 44) Two small single centre studies reported no significant differences in the incidence of adverse events between placebo and melatonin groups in patients with bipolar disorder(27) and major depressive disorder.(30) However, the methodology of adverse event reporting and classification of a serious adverse event were not defined in both studies.(27, 30) In patients with schizophrenia, melatonin was not associated with increased next day drowsiness, or occurrence of headache, heaviness of head and mental dullness compared to placebo.(33) No other adverse events were investigated, and the methodology of reporting of adverse events was not defined.(33) No adverse events were reported with three weeks of low dose (0.5 mg / day) melatonin in adults with ADHD and delayed sleep phase disorder,(36) however, the methodology of adverse event reporting and classification of a serious adverse event were not defined.

1. ***Management of insomnia in inpatients with substance use disorders.***

Summary of evidence: There is insufficient evidence to establish the role of melatonin in improving sleep quality among inpatients with substance use disorders. Available evidence of benefit is inconsistent.

Melatonin treatment has mixed results in improving sleep quality among patients with benzodiazepine dependence.(14, 45) Some randomised controlled trials have observed no beneficial effects with melatonin (daily dosing PR 2 mg or IR 5 mg, duration 24 weeks or 6 weeks) on objective sleep efficiency (i.e., total sleep time, sleep latency, number of awakenings, REM latency)(18) and subjective sleep quality(13) compared to placebo. Others have reported improved subjective sleep quality with melatonin (daily dosing PR 2 mg or IR 5 mg, duration ranged from 6 - 24 weeks) compared to placebo in patients withdrawing from long-term benzodiazepines.(18, 46, 47) In most studies evaluating benzodiazepine cessation or improvement in withdrawal symptoms, melatonin did not show any benefit over placebo.(14, 16, 19) A study that examined the effect of melatonin PR 2 mg/day for 12 weeks on cognitive functioning, quality of life, subjective well-being and psychosocial functioning during benzodiazepine withdrawal concluded no additional benefit over placebo.(17)

There is conflicting evidence on the role of melatonin for sleep quality in patients with opioid addiction. A randomised controlled trial in 54 patients receiving methadone treatment for opioid dependence observed improved perceived sleep quality, anxiety and depression in patients receiving melatonin IR 10 mg/day (for 12 weeks) compared to placebo.(48) Conversely, a cross-over study in 61 patients enrolled at a community methadone maintenance clinic attempting benzodiazepine withdrawal observed no difference in perceived sleep outcomes (sleep duration, sleep disturbances, sleep latency, daytime functioning due to sleepiness, sleep efficiency, sleep quality, sleep medication use) among those receiving melatonin IR 5 mg/day for 6 weeks compared to placebo.(13)

There is a paucity of data on the efficacy of melatonin for insomnia treatment in nicotine addiction or alcohol use disorder. A small cross-over study in 12 patients with nicotine addiction observed improved self-reported ratings of mood (e.g., anxious, irritable, angry, depressed) with a single oral dose of melatonin 0.3 mg administered after 3.5 hours of nicotine cessation.(49) In 60 patients with alcohol use disorder, melatonin IR 5 mg/day for 4 weeks did not show any improvement in self-reported sleep quality (measured using the Pittsburgh sleep quality index) in a small placebo-controlled trial.(50) Further studies are required to establish the role of melatonin for the management of insomnia in these settings.

Most studies investigating the use of melatonin in patients with benzodiazepine dependence, opioid addiction or alcohol use disorder did not explore the safety / tolerability of melatonin.(13, 14, 45, 48, 49) Small randomised controlled trials have reported good tolerability of melatonin, with a similar rate of adverse events to placebo and no serious adverse events.(16, 19, 47, 50) However, the methodology of adverse event reporting and classification of a serious adverse event were not defined.

## References

1. Lund Rasmussen C, Klee Olsen M, Thit Johnsen A, et al. Effects of melatonin on physical fatigue and other symptoms in patients with advanced cancer receiving palliative care: A double-blind placebo-controlled crossover trial. *Cancer*. 2015;121:3727-3736.

2. Del Fabbro E, Dev R, Hui D, et al. Effects of melatonin on appetite and other symptoms in patients with advanced cancer and cachexia: A double-blind placebo-controlled trial. *J Clin Oncol*. 2013;31:1271-1276.

3. Fan R, Bu X, Yang S, et al. Effect of melatonin on quality of life and symptoms in patients with cancer: A systematic review and meta-analysis of randomised controlled trials. *BMJ Open*. 2022;12:2022-060912.

4. Campbell AM, Axon DR, Martin JR, et al. Melatonin for the prevention of postoperative delirium in older adults: A systematic review and meta-analysis. *BMC Geriatrics*. 2019;19:272.

5. Khaing K, Nair BR. Melatonin for delirium prevention in hospitalized patients: A systematic review and meta-analysis. *J Psychiatr Res*. 2021;133:181-190.

6. Siddiqi N, Harrison JK, Clegg A, et al. Interventions for preventing delirium in hospitalised non-icu patients. *Cochrane Database Syst Rev*. 2016.

7. Ng KT, Teoh WY, Khor AJ. The effect of melatonin on delirium in hospitalised patients: A systematic review and meta-analyses with trial sequential analysis. *J Clin Anesth*. 2020;59:74-81.

8. Barnes J, Sewart E, Armstrong R, et al. Does melatonin administration reduce the incidence of postoperative delirium in adults? Systematic review and meta-analysis. *BMJ Open*. 2023;13:e069950.

9. Wibrow B, Martinez FE, Myers E, et al. Prophylactic melatonin for delirium in intensive care (pro-medic): A randomized controlled trial. *Intensive Care Med*. 2022;48:414-425.

10. Gandolfi JV, Di Bernardo APA, Chanes DAV, et al. The effects of melatonin supplementation on sleep quality and assessment of the serum melatonin in icu patients: A randomized controlled trial. *Crit Care Med*. 2020;48:e1286-e1293.

11. Aiello G, Cuocina M, La Via L, et al. Melatonin or ramelteon for delirium prevention in the intensive care unit: A systematic review and meta-analysis of randomized controlled trials. *J Clin Med*. 2023;12.

12. Bouajram R, Baumgartner L, Pham C, et al. 843: Efficacy of sleep medication administration and impact on delirium in critically ill patients. *Crit Care Med*. 2021;49.

13. Peles E, Hetzroni T, Bar-Hamburger R, et al. Melatonin for perceived sleep disturbances associated with benzodiazepine withdrawal among patients in methadone maintenance treatment: A double-blind randomized clinical trial. *Addiction*. 2007;102:1947-1953.

14. Wright A, Diebold J, Otal J, et al. The effect of melatonin on benzodiazepine discontinuation and sleep quality in adults attempting to discontinue benzodiazepines: A systematic review and meta-analysis. *Drugs Aging*. 2015;32:1009-1018.

15. Baandrup L, Ebdrup BH, Rasmussen J, et al. Pharmacological interventions for benzodiazepine discontinuation in chronic benzodiazepine users. *Cochrane Database Syst Rev*. 2018.

16. Baandrup L, Lindschou J, Winkel P, et al. Prolonged-release melatonin versus placebo for benzodiazepine discontinuation in patients with schizophrenia or bipolar disorder: A randomised, placebo-controlled, blinded trial. *World J Biol Psychiatry*. 2016;17:514-524.

17. Baandrup L, Fagerlund B, Glenthoj B. Neurocognitive performance, subjective well-being, and psychosocial functioning after benzodiazepine withdrawal in patients with schizophrenia or bipolar disorder: A randomized clinical trial of add-on melatonin versus placebo. *Eur Arch Psychiatry Clin Neurosci*. 2017;267:163-171.

18. Baandrup L, Glenthøj BY, Jennum PJ. Objective and subjective sleep quality: Melatonin versus placebo add-on treatment in patients with schizophrenia or bipolar disorder withdrawing from long-term benzodiazepine use. *Psychiatry Res*. 2016;240:163-169.

19. Lähteenmäki R, Puustinen J, Vahlberg T, et al. Melatonin for sedative withdrawal in older patients with primary insomnia: A randomized double-blind placebo-controlled trial. *Br J Clin Pharmacol*. 2014;77:975-985.

20. Lewis SR, Pritchard MW, Schofield‐Robinson OJ, et al. Melatonin for the promotion of sleep in adults in the intensive care unit. *Cochrane Database Syst Rev*. 2018.

21. Bellapart J, Appadurai V, Lassig-Smith M, et al. Effect of exogenous melatonin administration in critically ill patients on delirium and sleep: A randomized controlled trial. *Crit Care Res Pract*. 2020;23.

22. Jaiswal SJ, McCarthy TJ, Wineinger NE, et al. Melatonin and sleep in preventing hospitalized delirium: A randomized clinical trial. *Am J Med*. 2018;131:1110-1117.

23. Marupuru S, Arku D, Campbell AM, et al. Use of melatonin and/on ramelteon for the treatment of insomnia in older adults: A systematic review and meta-analysis. *J Clin Med*. 2022;11.

24. Grima NA, Rajaratnam SMW, Mansfield D, et al. Efficacy of melatonin for sleep disturbance following traumatic brain injury: A randomised controlled trial. *BMC Med*. 2018;16:017-0995.

25. Ferracioli-Oda E, Qawasmi A, Bloch MH. Meta-analysis: Melatonin for the treatment of primary sleep disorders. *PLoS One*. 2013;8.

26. Edemann-Callesen H, Andersen HK, Ussing A, et al. Use of melatonin in children and adolescents with idiopathic chronic insomnia: A systematic review, meta-analysis, and clinical recommendation. *EClinicalMedicine*. 2023;61.

27. Quested DJ, Gibson JC, Sharpley AL, et al. Melatonin in acute mania investigation (miami-uk). A randomized controlled trial of add-on melatonin in bipolar disorder. *Bipolar Disord*. 2021;23:176-185.

28. Bersani G, Garavini A. Melatonin add-on in manic patients with treatment resistant insomnia. *Prog Neuropsychopharmacol Biol Psychiatry*. 2000;24:185-191.

29. Livianos L, Sierra P, Arques S, et al. Is melatonin an adjunctive stabilizer? *Psychiatry Clin Neurosci*. 2012;66:82-83.

30. Dolberg OT, Hirschmann S, Grunhaus L. Melatonin for the treatment of sleep disturbances in major depressive disorder. *Am J Psychiatry*. 1998;155:1119-1121.

31. Serfaty MA, Osborne D, Buszewicz MJ, et al. A randomized double-blind placebo-controlled trial of treatment as usual plus exogenous slow-release melatonin (6 mg) or placebo for sleep disturbance and depressed mood. *Int Clin Psychopharmacol*. 2010;25:132-142.

32. Shamir E, Laudon M, Barak Y, et al. Melatonin improves sleep quality of patients with chronic schizophrenia. *J Clin Psychiatry*. 2000;61:373-377.

33. Suresh Kumar PN, Andrade C, Bhakta SG, et al. Melatonin in schizophrenic outpatients with insomnia: A double-blind, placebo-controlled study. *J Clin Psychiatry*. 2007;68:237-241.

34. Van der Heijden KB, Smits MG, Van Someren EJ, et al. Effect of melatonin on sleep, behavior, and cognition in adhd and chronic sleep-onset insomnia. *J Am Acad Child Adolesc Psychiatry*. 2007;46:233-241.

35. Weiss MD, Wasdell MB, Bomben MM, et al. Sleep hygiene and melatonin treatment for children and adolescents with adhd and initial insomnia. *J Am Acad Child Adolesc Psychiatry*. 2006;45:512-519.

36. van Andel E, Bijlenga D, Vogel SWN, et al. Effects of chronotherapy on circadian rhythm and adhd symptoms in adults with attention-deficit/hyperactivity disorder and delayed sleep phase syndrome: A randomized clinical trial. *Chronobiol Int*. 2021;38:260-269.

37. Williams Buckley A, Hirtz D, Oskoui M, et al. Practice guideline: Treatment for insomnia and disrupted sleep behavior in children and adolescents with autism spectrum disorder: Report of the guideline development, dissemination, and implementation subcommittee of the american academy of neurology. *Neurology*. 2020;94:392-404.

38. Abdelgadir IS, Gordon MA, Akobeng AK. Melatonin for the management of sleep problems in children with neurodevelopmental disorders: A systematic review and meta-analysis. *Archives of disease in childhood*. 2018;103:1155-1162. Epub 2018/05/04.

39. McDonagh MS, Holmes R, Hsu F. Pharmacologic treatments for sleep disorders in children: A systematic review. *J Child Neurol*. 2019;34:237-247.

40. Gringras P, Nir T, Breddy J, et al. Efficacy and safety of pediatric prolonged-release melatonin for insomnia in children with autism spectrum disorder. *J Am Acad Child Adolesc Psychiatry*. 2017;56:948-957.

41. Maras A, Schroder CM, Malow BA, et al. Long-term efficacy and safety of pediatric prolonged-release melatonin for insomnia in children with autism spectrum disorder. *J Child Adolesc Psychopharmacol*. 2018;28:699-710.

42. Schroder CM, Malow BA, Maras A, et al. Pediatric prolonged-release melatonin for sleep in children with autism spectrum disorder: Impact on child behavior and caregiver's quality of life. *J Autism Dev Disord*. 2019;49:3218-3230.

43. Malow BA, Findling RL, Schroder CM, et al. Sleep, growth, and puberty after 2 years of prolonged-release melatonin in children with autism spectrum disorder. *J Am Acad Child Adolesc Psychiatry*. 2021;60:252-261.

44. Galli-Carminati G, Deriaz N, Bertschy G. Melatonin in treatment of chronic sleep disorders in adults with autism: A retrospective study. *Swiss Med Wkly*. 2009;139:293-296.

45. Das A, Prithviraj M, Mohanraj PS. Role of melatonin in the management of substance addiction: A systematic review. *Cureus*. 2022;14.

46. Garfinkel D, Zisapel N, Wainstein J, et al. Facilitation of benzodiazepine discontinuation by melatonin: A new clinical approach. *Arch Intern Med*. 1999;159:2456-2460.

47. Garzón C, Guerrero JM, Aramburu O, et al. Effect of melatonin administration on sleep, behavioral disorders and hypnotic drug discontinuation in the elderly: A randomized, double-blind, placebo-controlled study. *Aging Clin Exp Res*. 2009;21:38-42.

48. Ghaderi A, Banafshe HR, Mirhosseini N, et al. The effects of melatonin supplementation on mental health, metabolic and genetic profiles in patients under methadone maintenance treatment. *Addict Biol*. 2019;24:754-764.

49. Zhdanova IV, Piotrovskaya VR. Melatonin treatment attenuates symptoms of acute nicotine withdrawal in humans. *Pharmacol Biochem Behav*. 2000;67:131-135.

50. Gendy MNS, Lagzdins D, Schaman J, et al. Melatonin for treatment-seeking alcohol use disorder patients with sleeping problems: A randomized clinical pilot trial. *Sci Rep*. 2020;10:020-65166.
